# Takotsubo Cardiomyopathy: Typical and Atypical Variants, A Two-Year Retrospective Cohort Study

**DOI:** 10.14740/cr349w

**Published:** 2014-10-06

**Authors:** Avinash Murthy, Jaspreet Arora, Arti Singh, Maheedhar Gedela, Pavan Karnati, Anthony Nappi

**Affiliations:** a Division of Cardiology, Bridgeport Hospital, 267 Grant Street, Bridgeport, CT 06484, USA; b Department of Internal Medicine, Albany Medical Center, 47 New Scotland Avenue, Albany, NY 12208, USA; c Division of Cardiology, Department of Internal Medicine, Albany Medical Center, 47 New Scotland Avenue, Albany, NY 12208, USA; d Department of Medicine, Clinch Valley Medical Center, 6801 Gov. G. C. Peery Highway, Richlands, VA 24641, USA

**Keywords:** Takotsubo cardiomyopathy, Atypical takotsubo cardiomyopathy, Stress cardiomyopathy, Classical takotsubo cardiomyopathy

## Abstract

**Background:**

Typical or classical takotsubo cardiomyopathy (TCM) is associated with the characteristic abnormality of a ballooned left ventricular apex with basal segmental hyperkinesis. TCM may not present with the “classical” wall motion abnormalities but can have a variety of segmental wall motion abnormalities. The aim of our work was to assess for any unique identifying factors that can help distinguish typical and atypical variants of TCM.

**Methods:**

We studied 11 consecutive patients between 2010 and 2012 admitted with chest pain, electrocardiographic and cardiac biomarker changes consistent with acute coronary syndrome (ACS) who underwent left heart angiography and were clinically diagnosed to have TCM.

**Results:**

Our study found no specific features distinguishing typical and atypical variants of TCM. In our study, all patients were female and all had excellent outcome. One patient was in fourth decade of life, three patients in fifth and sixth decade of life, while remaining were older. One patient had diabetes mellitus, five had hypertension, four had concurrent coronary artery disease, but no patient had any family history of TCM. Nine of 11 patients had immediate clear-cut stressors. Three patients had normal ECG, two with ST segment elevation, with nine patients having only modest troponin elevations. One patient had an anomalous RCA take-off from the right coronary cusp, otherwise remaining patients had normal anatomy. One patient had only apical involvement, remaining had multiple wall motion abnormalities, and all patients had involvement of the anterior wall. Four patients had apical sparing. No inverted TCM pattern with basal akinesis with normal wall motion in the midventricular and apical regions was found among our patients.

**Conclusions:**

We report that the classification of TCM as typical versus atypical is probably not clinically meaningful. The regional wall motion abnormalities are related to catecholamine excess and to the susceptibility of that particular region to excess catecholamine. We do not know why such differences in regional susceptibility exist, and agree with the other authors that sub-classification would only add to confusion, and a delay in understanding of the disease process.

## Introduction

“Takotsubo cardiomyopathy” (TCM) or “broken heart syndrome” or “stress cardiomyopathy” is frequently applied to presentations of transient wall motion abnormality in the absence of a culprit epicardial coronary artery lesion. It had been typically associated with the characteristic abnormality of a ballooned left ventricular apex with basal segmental hyperkinesis. As we become more aware and report more cases of patent coronary artery in the setting of acute coronary syndrome (ACS), we are beginning to learn that TCM need not necessarily present with the “classical tako-tsubo” type wall motion abnormalities but can rather have a variety of segmental wall motion abnormalities; these presentations are termed “atypical” variants of TCM.

## Patients and Methods

We studied “atypical” variants of TCM presenting to Albany Medical Center over a 2-year period, with a goal to report any unique identifying factors differentiating the “typical” and “atypical” variants of TCM. We studied 11 consecutive patients between years 2010 and 2012 admitted with chest pain, electrocardiographic changes consistent with ACS who underwent left heart angiography and were clinically diagnosed to have TCM. Retrospective chart review was performed. Demographics, clinical, echocardiographic, angiographic data and management plan were collected.

## Results

Based on our data analysis, we were not able to identify any differentiating factors responsible for patients presenting with typical or atypical variants of TCM. The outcomes were excellent in all the patients. In our study, four of our patients did not have apical involvement (Table 1), suggesting that up to 40% may have apical sparing.

**Table 1:** Imaging Data and Management

Patient	% EF by LV Gram	Regional wall motion abnormality by angiography	Angiographic findings	Management
1	20	Anteroapical, inferoapical	Normal coronaries	ACE, BB, spironolactone, digoxin
2	15 - 20	Anteroapical, inferoapical, anterolateral	Normal coronaries	ASA, ACE, BB, statin
3	45	Mid anterior	Normal coronaries	ASA, ACE, BB
4	35	Circumferential	Normal coronaries	ASA, ACE, BB, statin
5	45	Anteroapical	30% Prox LAD stenosis	ASA, dalteparin, coumadin
6	40 - 45	Midanterior	Anomalous take off of RCA, normal coronaries	ASA, plavix, ACE, BB, statin
7	35	Midanterior, apical, midinferior	Patent LAD stent, patent coronaries	ASA, ACE, BB
8	< 25	Anterior, anteroapical, inferoapical, midinferior	50% RCA 1 lesion, LAD 50% 2 lesions, second diagnol 50% lesion	ASA, BB, statin
9	45	Midanterior, apical, midinferior	Normal coronaries	Plavix, ACE, BB, statin
10	40	Apical	Occluded marginal, otherwise normal	ASA, plavix, ARB, BB, statin
11	45	Midanterior, midinferior	Normal coronaries	BB, ACE

In our study, all subjects were female, with the youngest patient in the fourth decade, three subjects in the fifth and sixth decade of life, and the remainder of the study patients being older (Table 2). Among our case series, only one had diabetes mellitus and five patients had a history of hypertension. Four of the patients were cigarette smokers, no significant inference about the co-morbidities and its association with either typical or atypical variants of TCM could be drawn among our patients (Table 2).

**Table 2:** Patient Demographics

Patient	Age	Sex	Time of onset of symptoms	DM	HTN	Smoking	Stressor
1	64	F	AM	N	Y	Y	COPD exacerbation
2	76	F	AM	N	Y	N	PNA
3	53	F	PM	N	N	N	Family issues
4	81	F	PM	N	Y	Y	Strenuous physical activity
5	43	F	PM	N	N	Y	Chemotherapy
6	65	F	PM	N	N	N	Strenuous physical activity
7	54	F	PM	N	N	Y	Unknown, possibly alcohol
8	72	F	NA	Y	Y	N	Unknown, possibly anxiety
9	67	F	PM	N	N	N	Fall from scooter
10	77	F	AM	N	Y	N	Family issues
11	51	F	NA	N	N	N	Strenuous physical activity

Nine of our patients had immediate clear-cut stressors and two of them could not identify any significant stressors, although one of them had substantial alcohol consumption. All of our patients were female and we were not able to accurately determine their menopausal state based on our records. We did not find any family history of TCM and there was no mention of any genetic diseases.

In our cohort, the ECG was normal in three patients, with ST segment elevation only present in two patients. Most of our patients had modest troponin elevations with the exception of two patients, one of whom presented with ST segment elevations of the ECG (Table 3). One of our patients had an anomalous RCA take-off from the right coronary cusp. Otherwise, none of the patients in our study demonstrated any of anatomic abnormalities.

**Table 3:** Lab Data

Patient	Peak troponin (ng/mL)	Peak CK (ng/mL)	Peak CKMB%	Admit EKG	Admit QTc on EKG (ms)	Discharge QTc on EKG (ms)
1	1.45	131	9.2	TWI V3-V6, & AvL, non-specific ST changes	478	438
2	10.59	2,562	3.3	STEMI I, aVL, V2-3	400	436
3	2.92	178	6.2	TWI I, aVL, V1	416	443
4	7.37	400	13	TWI V2-V6, I, aVL, non-specific ST changes	415	405
5	0.7	60	14.2	TWI I, II, aVF, V4-6	501	492
6	2.7	17	10.5	Normal	435	444
7	1.76	168	5.8	Prolonged QT	480	534
8	0.33	32	13	Atrial paced, frequent PVC, lateral TWI, questionable anterior STEMI	486	462
9	2.18	107	17.6	NSR with first AVB, low voltage	432	469
10	1.86	512	1.9	Normal, low voltage	428	430
11	1.03	89	8	Normal	443	470

Four of our patients had concurrent coronary artery disease (CAD), which were deemed as not to be responsible for the wall motion abnormalities that were noted. In one patient, only the apex was involved, as described in typical variant, all the others had multiple wall motion abnormalities, and all of them involved the anterior wall, either anteroapical or/and midanterior segments. Inferoapical, midinferior walls were also frequently involved. While inferoapical had concurrent anteroapical segmental wall motions, the midinferior segments had concurrent midanterior segmental wall motion abnormalities. We did not find any inverted TCM in our patients where there is basal akinesis with normal wall motion in the midventricular and apical regions.

## Discussion

Given that there was no difference in patient presentation and outcomes, we suggest that it is not meaningful to classify TCM patients into further subgroups. TCM is a syndrome in which there is a characteristic transient myocardial wall motion abnormality in the absence of obstructive CAD or acute plaque rupture. The left ventricular wall motion abnormalities usually extend beyond a single epicardial vessel distribution. Patients with this condition will present with ECG changes and/or serum biomarker elevations [1].

In 2004, The Mayo Clinic proposed diagnostic criteria for TCM: 1) There is transient hypokinesis, akinesis, or dyskinesis of the mid segments of the left ventricle (LV) with or without apical involvement and the regional wall motion aberrations spread beyond a single epicardial vascular distribution. 2) There is absence of obstructive CAD or evidence of acute plaque rupture evidence angiographically. 3) There are new EKG abnormalities or modest rise in troponin. 4) There is absence of myocarditis and/or pheochromocytoma [2].

The Japanese first coined the term “takotsubo” in 1991 after an octopus trap. These traps have a round bottom with a narrow neck, reminiscent of the characteristic finding on a left ventricular angiogram in a patient with the syndrome. Other nomenclatures such as “stress-induced” cardiomyopathy, “broken heart” syndrome, and “apical ballooning” syndrome were used to describe the condition. As the condition has become more recognized, there have been more reports and the emergence of more “atypical” forms. While the “typical” TCM is described as systolic contractile dysfunction involving the mid and apical segments of the LV keeping in shape with the “takotsubo”, the atypical forms usually spare the apex of the heart and involve the midventricular or basal segments. In a study among troponin positive ACS patients, Kurowski et al described an incidence of 1.2% transient cardiomyopathy, among which only 60% of subjects had “typical” TCM features. The remaining 40% of the patients in that cohort had “atypical” variants [3]. There are no differences reported in the demographics, clinical findings, angiographic findings, laboratory parameters or disease outcomes among patients with TCM “variants” [4-6].

The Mayo Clinic diagnostic criteria for the diagnosis of TCM interestingly and correctly, do not include involvement of apex as one of its diagnostic criteria [2]. In our study, four of our patients did not have apical involvement (Table 1), suggesting that up to 40% may have apical sparing. These findings are comparable to a previous study by Kurowski et al [3]. Both typical and atypical variants of TCM are predominantly found in post-menopausal women aged 62 - 76 years, and the reasoning behind the gender difference is not well understood [7]. In our study, all subjects were female, with the youngest patient in the fourth decade, three subjects in the fifth and sixth decade of life, and the remainder of the study patients being older (Table 2). The presence or absences of co-morbidities were not well studied in these populations, although any acute illness or medications can possibly trigger stress cardiomyopathy. Among our case series, only one had diabetes mellitus and five patients had a history of hypertension. Four of the patients were cigarette smokers, and no significant inference about the co-morbidities and its association with either typical or atypical variants of TCM could be drawn among our patients (Table 2).

A significant emotional or physical stressor typically precedes the development of TCM. Male patients have predominantly physical stressors, whereas women with TCM have predominantly emotional or no identifiable stressor at all [7]. However, inability to identify a stressor does not preclude the disease, as one-third of TCM patients are without a precipitating factor, and the Mayo Clinic criteria do not include the presence of a stressor for diagnosing TCM as stated above. Nine of our patients had immediate clear-cut stressors and two of them could not identify any significant stressors, although one of them had substantial alcohol consumption. While the association between stress, whether emotional or physical, and TCM is not fully understood, it is hypothesized that it is mediated through stress-induced catecholamine surge [8]. Similar clinical pictures and cardiomyopathies have been seen in patients with pheochromocytoma and those receiving exogenous epinephrine or dobutamine. Epinephrine or norepinephrine can engage adrenergic receptors leading to direct myocardial injury. It is hypothesized that some of them are more susceptible to these hormones/stress because of genetic differences [9]. Our study did not involve the study of any such genetic differences. Differences in myocardial adrenergic receptor density or distribution, or estrogen depletion, have also been implicated [10]. All of our patients were female and we were not able to accurately determine their menopausal state based on our records. Recent advances in DNA sequencing and exome capture have given us the opportunity to study possible genetic predispositions to TCM, and also genetic diseases, such as Fragile X and long QT syndrome, associated with TCM [9]. We did not find any family history of TCM and there was no mention of any genetic diseases. Efforts to study temporal relationship with the timing of onset of TCM, reflecting the endogenous levels of cortisol and catecholamines have not reached consensus due to the limited amount of data [11]. In our patients, the presentation to the ER/symptom onset did not have any temporal relationship with the offending stressor.

The most common findings on ECG are ST segment elevations and T wave inversions. Up to 81% of TCM patients will have ST segment elevations, and up to 64% will have T wave inversions. Compared with AMI, the ST segment elevation magnitudes in TCM are smaller, and the elevations are usually found in the precordial leads. There also may be a transient QT prolongation, which most often resolves within 24 - 48 h. Other arrhythmias can occur, but are less common [12]. In our cohort, the ECG was normal in three patients, with ST segment elevation only present in two patients. Elevation of cardiac biomarkers can be found in TCM with troponin elevations in 85%, creatinine kinase elevations in 53%, and creatinine kinase-MB elevations in 38%. The degrees of the elevations are usually less than with patients who have an STEMI, but similar to that in non-ST elevation ACS. Most of our patients had modest troponin elevations with the exception of two patients, one of whom presented with ST segment elevations of the ECG (Table 3).

Other less popular theorized causes of TCM include anatomical coronary alterations, coronary vasospasm, and left ventricular outflow tract obstruction [13-15]. One of our patients had an anomalous RCA take-off from the right coronary cusp. Otherwise, none of the patients in our study demonstrated any of the above mentioned abnormalities. Angiography shows wall motion abnormalities in the absence of significant CAD to explain the distribution of the wall motion abnormalities. Of note, patients with TCM can have concurrent CAD, but not in the distribution of the akinesis, as many of the patients with TCM are post-menopausal women who also have risk factors for CAD [16]. Four of our patients had concurrent CAD, which were deemed as not to be responsible for the wall motion abnormalities that were noted. In one patient, only the apex was involved, as described in typical variant, all the others had multiple wall motion abnormalities, and all of them involved the anterior wall, either anteroapical or/and midanterior segments. Inferoapical, midinferior walls were also frequently involved. While inferoapical had concurrent anteroapical segmental wall motions, the midinferior segments had concurrent midanterior segmental wall motion abnormalities. We did not find any inverted TCM in our patients where there is basal akinesis with normal wall motion in the midventricular and apical regions.

Myocarditis or pheochromocytoma can mimic TCM. Although we were able to clinically exclude pheochromocytoma, we did not perform cardiac MRI to rule out myocarditis in our cases. Nonetheless, the rapid in-hospital clinical recovery pointed toward a diagnosis of TCM rather than myocarditis in all of our patients. Beta-blockade may counter excess catecholamine levels. Although no randomized trials have been performed, treatment with beta-blocker has been part of mainstay therapy. Patients with systolic dysfunction should also receive an ACE inhibitor (ACE-I). However, there is less evidence for ACE-I use in TCM as shown by retrospective studies. Nevertheless, some retrospectives studies have shown that treatment with ACE-I protects against adverse outcomes after TCM diagnosis. Of note, about 20% of patients with TCM have presented while already being treated with beta-blocker or ACE-I for other pathologies such as hypertension. Aspirin and clopidogrel are not recommended based on the fact that TCM is not due an ACS [17]. Anticoagulation is recommended in patients with TCM and a left ventricular thrombus. All of our patients, except one received beta-blockers and ACE-Is and did well upon discharge.

TCM generally carries an excellent prognosis if no complications occur during the early infarction period and myocardial function can be expected to return to normal within a few weeks or less [18]. Complications occur in 20% of TCM cases and include the following: left heart failure with and without pulmonary edema, cardiogenic shock, left ventricular obstruction, mitral regurgitation, ventricular arrhythmias, left ventricular mural thrombus formation, left ventricular free wall rupture and death [19, 20]. There is one particular studied high-risk subgroup in which TCM patients with severe acute heart failure and hypotension can have a hospital mortality rate of up to 20% despite aggressive medical intervention [21]. Nonetheless, prognosis is generally excellent with nearly 95% of patients experiencing complete recovery within 4 - 8 weeks. Recurrence rates vary, but it is estimated at 3% [22]. All of our patients did well until discharge, with only one recurrence reported back to our institution. Since our center is a referral center, we were not able to obtain follow-up echocardiograms of all the patients to ascertain long-term outcomes.

### Conclusion

We found no specific features like EKG, LV angiogram, troponin elevation, or associated co-morbidity specific to either typical or atypical variants of TCM. We did not notice any influence of circadian rhythm or specific age patterns causing atypical versus typical TCM, though all of our cases were amongst females, with majority of them being menopausal. All cases had a general theme in that the distribution of myocardial wall motion abnormality did not follow along with the coronary flow distribution and prognosis was generally good with medical therapy. We present our experience with 11 cases of TCM from our center. Our series suggests that classification of TCM as typical versus atypical is probably not clinically meaningful and that there are no significant differentiating factors between these two variants. We believe that in TCM, there is catecholamine excess and the characteristic regional wall motion abnormality is related to the susceptibility of that particular region to excess catecholamine. We do not know why such differences in susceptibility exist. We agree with the other authors that sub-classification would only add to confusion, and a delay in understanding of disease process [6].

### Learning objectives

1) Atypical variants of TCM are common. 2) No unique identifying factors distinguishing typical and atypical variants. 3) Prognosis is good in both variants of TCM. 4) Classification of TCM into typical and atypical variants is probably clinically less meaningful.

## Author Note

The work was performed at the Division of Cardiology, Albany Medical Center, 47 New Scotland Avenue, Albany, NY 12208, USA.
